# Redesigning online research methodology education: insights from undergraduate rehabilitation students' experiences in South Africa

**DOI:** 10.1186/s12909-025-06934-0

**Published:** 2025-03-31

**Authors:** Quinette Abegail Louw, Karina Berner, Alida De Beer, Maria Yvonne Charumbira, Conran Joseph

**Affiliations:** 1 Division of Physiotherapy, Department of Health and Rehabilitation Sciences, Faculty of Medicine and Health Sciences, Stellenbosch University, Cape Town, 7500 South Africa; 2 Division of Speech, Language and Hearing Therapy, Department of Health and Rehabilitation Sciences, Faculty of Medicine and Health Sciences, Stellenbosch University, Cape Town, 7500 South Africa

**Keywords:** Online education, Rehabilitation, Undergraduate research

## Abstract

**Background:**

Online education has become increasingly prevalent in higher education. However, there are insufficient research-led pedagogies to inform faculty aspiring to develop an online rehabilitation research methodology curriculum. This study aimed to explore a South African university’s undergraduate rehabilitation students' experiences with a newly developed online research methodology module.

**Methods:**

A cross-sectional mixed-methods survey was conducted using a questionnaire with a mix of Likert scales and open-ended questions. This paper reports qualitative feedback from three undergraduate rehabilitation student cohorts. Thematic analysis in Atlas.ti.22.2.4 used an abductive approach.

**Results:**

Forty-seven third-year Physiotherapy students in 2021 (response rate = 100%), 46 fourth-year Physiotherapy students in 2022 (response rate = 43%), and 26 third-year Speech-Language and Hearing Therapy students in 2023 (response rate = 38%) provided feedback. Key themes related to engagement and communication, content, and online user interfaces emerged. The findings highlighted the importance of dedicated support, clear communication about feedback, structured peer interactions, a clear and structured presentation of content aligned with explicit outcomes and explicit links between course components and professional practice in online research methodology courses. A well-organised, intuitive user interface and timely availability of engaging learning materials are crucial.

**Conclusion:**

The findings underscore several critical aspects of effective online rehabilitation research methodology education. Addressing identified gaps, such as improving systematic feedback and clarifying task integration, can enhance the online research methodology learning experience for undergraduate rehabilitation students. The insights provided may be considered in the design of similar online medical courses.

**Supplementary Information:**

The online version contains supplementary material available at 10.1186/s12909-025-06934-0.

## Background

The landscape of higher education is evolving, with online teaching emerging as a prominent mode of instruction [1]. Online education is defined as the delivery of educational content and instruction through the internet or other digital technologies [2]. This shift towards online education was accelerated by the global pandemic, which demanded the rapid development of entirely online delivery of courses and assessments for continued education [3]. Transitioning to online modes facilitated the acceptance and familiarity of online education by educators and students, largely due to increased exposure and repeated interactions with online tools [4]. The rapid increase in online education and acceptance rates has increased to the extent that it is now perceived as equal to or preferred over face-to-face teaching [4]. However, despite the increased use and acceptance of online education in higher education, there are insufficient research-led pedagogies for innovative teaching methods in undergraduate research methodology courses.

Research-informed innovation is crucial for online research methodology courses [5]. Students, especially undergraduate students in applied fields such as rehabilitation, perceive research methodology as a daunting module, even when they use face-to-face teaching modes. Student perceptions are mirrored in educators, who find these modules challenging and complex to teach. Transitioning towards online education requires evidence to inform the online development of research methodology, which involves the consideration of “what” needs to be taught as well as “how” it should be delivered to ensure engagement and connectedness. Recognising that learning within a virtual context is different from learning within face-to-face settings, teaching strategies need to be adapted to encourage deep, meaningful, and enjoyable learning experiences tailored to the online environment.

Research methodology courses require high levels of teacher‒student and peer interaction, support and mentoring during the research process to deliver high-quality research outputs and competent graduates [6]. Rehabilitation students often fail to comprehend the rationale and linkage between the clinical and research modules. The online learning environment adds another layer of complexity for students and staff when research methodology is taught online [7]. Staff also report insufficient motivation and inadequate knowledge, skills and uncertainties regarding students' preparedness and capabilities in online undergraduate research courses [8]. These challenges necessitate the need for evidence-informed approaches to ensure that rehabilitation students are equipped with the professional competencies required for professional practice and growth [9].

Skills development in an online research methodology course can encourage graduates to explore innovative technologies and research informed practices [10, 11]. An online research methodology course allows flexible and accessible participation from spatially diverse students, including students from other programs, to encourage collaborative, multidisciplinary practice. Online learning with peers can also positively influence work-related skills such as digital communication, which are increasingly relevant in remote and telerehabilitation practices [12]. The benefits of an online mode methodology course can thus potentially better prepare students for lifelong use of online methods to translate research for better patient care.

Understanding how students perceive and prefer to interact with online research methodology courses is crucial for optimal learning and acceptance. Existing information on the design of online courses offers valuable insights into elements that can enrich the learning experience for students. Key design aspects, such as the user-friendliness of the online platform and intuitive, easy-to-follow prompts, have been reported to positively impact the learning experience. Content can be presented in various modalities, including providing a variety of instructional materials, chunking content into manageable segments, and offering clear instructions [13]. A commonly reported disadvantage of online learning is the lack of connectedness to the educator, course material and peers, which can restrict the overall learning experience [1]. Therefore, interactions for structured engagement and communication are critical components of online courses. Strategies to enhance these elements include providing opportunities for student-to-student interaction and including collaborative activities to support active learning [13]. Additionally, incorporating activities designed to build a sense of community among peers is essential for fostering a supportive online learning environment.

To our knowledge, there are no published reports on undergraduate rehabilitation students' perceptions of an online research methodology course that could inform faculty aspiring to present research methodology online. This lack of evidence may stifle innovation and prevent students from benefiting from the flexibility, accessibility, and convenience of online education. This study aimed to explore a South African university’s undergraduate rehabilitation students' experiences with a newly developed online research methodology module. The findings of this study have the potential to inform the development and refinement of online research methodology courses, not only in rehabilitation sciences but also in related health profession education programs.

## Methodology

The cross-sectional mixed-methods survey, conducted between 2021 and 2023, formed part of the routine quality assurance of a newly developed online research methodology training module that has been implemented within Stellenbosch University’s (SU) Department of Health and Rehabilitation Sciences since 2021. The rationale for research training revision and the design of the new module has been reported in detail elsewhere [14]. Briefly, the revised Research Methodology (RM) module was developed to foster interprofessional collaboration among undergraduate students from Physiotherapy (PT), Occupational Therapy (OT), and Speech-Language and Hearing Therapy (SLHT) programs. Utilizing the Knowledge to Action Framework, the development process involved securing leadership support, conducting a scoping review to identify essential research competencies, and analyzing existing course content across divisions. This comprehensive approach led to the establishment of core undergraduate rehabilitation research competencies and the design of a shared learning environment emphasizing knowledge translation. Recognizing that undergraduate students may not have the capacity or time to fully understand multiple study designs, the module focuses on equipping students with a foundational understanding and unbiased overview of various research designs and approaches (both quantitative and qualitative).

The module extends over two years and was presented to third-year Physiotherapy (PT) division students in the second semester of 2021, extending into their fourth (final) year in 2022. In addition, selected topics were incorporated into the Division of Speech-Language and Hearing Therapy’s (SLHT) third-year research module (July–September 2023). For this research, we were especially interested in students’ experiences with a fully online module presentation mode. Table 1 presents the module’s design features and associated principles and content.

**Table 1:** Key design features of the Research Methodology module

Design feature of new module	Principles and content
Soft design features	1. Methodological pluralism: Embracing both quantitative and qualitative research methodologies, focusing on core research pillars rather than specific study designs 2. Conceptual understanding: Emphasis on understanding the 'what and why' of research concepts rather than just the 'how to', promoting critical thinking and deeper comprehension 3. Clinical relevance: Aligning content closely with clinical practice, using engaging and easy-to-follow elements to bridge the gap between abstract research concepts and practical application 4. Innovative teaching methods: Employing narratives and fun elements (e.g., 'the best holiday ever' for research questions) to make complex concepts more accessible and engaging 5. Authentic assessment: Using a case study approach with real patients from clinical rotations, providing a realistic context for applying research skills 6. Scaffolded learning: Designing content to build upon previous sessions, allowing multiple opportunities to revisit and apply key competencies throughout the module 7. Reflective practice: Incorporating activities that promote critical thinking, problem-solving, and self-reflection to develop higher-order thinking skills
Asynchronous, self-directed	Students engage with short podcasts, videos, and interactive activities at their own pace, yet structured with set synchronous check-in points and submission deadlines for activities. Formative assessments provide feedback, while project work is conducted in small groups. This approach offers flexibility for students to manage their learning independently within a structured framework
Fully online	Content is delivered via the SUNLearn learning management system, with synchronous sessions held by videoconferencing (e.g., MS Teams). Assignments are submitted online, and electronic feedback is provided to students
Learning site and activities	• SUNLearn online platform for asynchronous content and activities • MS Teams for synchronous sessions • Activities include short podcasts, interactive exercises, forum posts, quizzes, case-based activities, guided practice, scientific writing workshops, and group project work • Assessments include case planning, argumentative essay, case report draft, search strategy development, and final project (case plus literature review) manuscript and oral presentation • Incorporation of library training for database searching • Use of peer assessment and self-reflection activities

### Sample

The sample consisted of all Bachelor of Science (BSc) Physiotherapy students enrolled in the Research Methods 372 (RM 372) module in 2021 (third-year students) and the Research Methods 472 (RM 472) module in 2022 (fourth-year students). The research methods course is offered over two years in the Physiotherapy program (3rd and 4th years). Thus, new content is presented in the 4th year. All Bachelor (B) of Speech-Language and Hearing Therapy students enrolled in SPH 364 (third-year students): Research as a Professional Function module in 2023, were also invited to participate, constituting a census approach. There were thus no exclusion criteria, apart from not voluntarily participating. This approach ensured comprehensive coverage of student experiences with the new online module, although we considered that response rates would vary across questionnaires. The use of routinely collected data allowed for a naturalistic capture of student feedback, potentially reducing response bias associated with targeted research surveys while also acknowledging the common challenge of low response rates in educational feedback processes.

### Data collection instruments

The survey instrument used in this study was adapted from the standardised questionnaire developed by Stellenbosch University's Centre for Teaching and Learning (CTL) for undergraduate module evaluation [15], with modifications to suit the specific needs of the Research Methods module (Supplementary file 1). While the standard CTL survey has been in use for many years and has undergone iterative development, information about formal validation studies for this specific instrument was not available in the reviewed documentation. The questionnaire covered various aspects, including students' overall impressions of the learning experience, engagement with course material, and evaluation of specific topics (such as highlights and frustrations and level of interest in the content). It employs a mix of Likert-scale questions, ranging from 'strongly disagree' to 'strongly agree', and open-ended questions to capture qualitative feedback. The key themes addressed included student interest in research, pace and structure of the module, clarity of learning outcomes, content presentation, learning activities, online engagement, workload, and confidence in applying learned concepts. The feedback form was structured to include sections for overall module evaluation and topic-specific feedback. Two versions were used to obtain feedback over the course of the module: a comprehensive form containing both sections and a shorter version containing only the topic-specific feedback section. While this tailored approach allows for module-specific evaluation according to a standardised form, it is important to acknowledge potential limitations such as the timing of administration and the risk of questionnaire fatigue [16]. The shift to an electronic format by the CTL in recent years has improved accessibility and data processing efficiency but may have implications for response rates that need to be considered when interpreting results.

In this paper, we report on the responses to open-ended questions only, specifically those relating to the perceptions of students on the online mode, functionality, experience and preferences (Table 2).

**Table 2:** Open-ended survey questions concerning students’ experience with the new research training module

Question	Short (per-topic)/long (overall) form survey
**Overall impressions**	
What is one thing that you **enjoyed the most** [*about today’s session(s)/this semester*] and why?	Long and short
What did you find to be the most **frustrating** and why?	Long and short
Any **additional comments** on these lectures and activities—including ideas on how we may improve?	Short
Was the **order** and/or **structure** and/or **time allocation** of the different sessions (including breaks) beneficial for you to be able to fully participate and/or complete tasks? Why do you say that?	Long
Did you require more information in the initial introductory session to the module? If so, what sort of information would you have liked to hear about?	Long
What did you think of the **lecture podcasts** (e.g. would have liked more/less detail/depth/time, etc.)	Long
Any comments on the [*YouTube*] **videos**?	Long
Did the content and nature of the [*practical application*] **activities** allow you to practice and better understand what you have learned about in the session? Briefly explain your answer	Long
Did you find the way that **feedback** was provided on various activities [*and assessments*] sufficient? If not, please explain and give suggestions for improvement	Long
What did you think of the following **Assessment** opportunity [*add opportunity*]? Please comment on the (i) instructions, (ii) rubric, (iii) allocated time, (iv) relevance to the course, (v) usefulness as a learning opportunity and (vi) anything else you want to comment on	Long
**Engaging with the course page and material/online experience**	
What did you think of the **layout and design** of the SUNLearn [*module*] page (e.g. too busy, easy to navigate, confusing, too many links, etc.)?	Long
How did you **access** the course (e.g. laptop/mobile device)? Did you have any concerns regarding data usage (e.g. when leaving SUNLearn to link to YouTube)?	Long
Did you find it easy to **connect with other students** (e.g. group work, sharing resources, ideas)? Why or why not?	Long
**Per topic session/lecture**	
Please **rate** the topic/session “[*add title*]” by giving it a mark from 0–10 ("0" meaning you gained nothing from the session versus "10" meaning you learnt maximally from the session). Please **motivate** your answer	Short
Any **additional comments** on this session? (optional)	Short
**Final thoughts**	
Do you have any other comments/feedback for this module? (optional)	Long

Text in square brackets was customised per survey based on the survey's timing/topic or has been added here for context (some questions were preceded by related Likert scale items introducing the focus of the subsequent open-ended question)

### Data collection procedures

As part of quality assurance and to inform the ongoing module development, PT students provided anonymous session feedback via SU’s existing online Learning Management System (SUNLearn) throughout the module course. The students also had the option to complete feedback in hard copy form (this format was ultimately only used by the SPH students). For paper-based submissions, questionnaires and consent forms were submitted separately. The forms were completed by students voluntarily on an ongoing basis from the implementation of the module in June 2021. The comprehensive form (20–30 min to complete) was administered at the start and end of the third-year PT module in 2021 and at the end of the fourth-year PT module (2022). The shorter topic-specific form (5–10 min to complete) was administered throughout the module after individual topics were covered in the respective years. SPH students were similarly asked to provide anonymous feedback on the topics presented to them.

### Ethical considerations

Ethical clearance was obtained from the SU Health Research Ethics Committee (N21/11/130), both for including feedback routinely obtained prior to the ethics approval date (2021) and for continuing to collect data prospectively (2022–3). Survey participation was voluntary and anonymous. Each survey was accompanied by an informed consent form, and for online versions, clicking agreement to complete the survey served as informed consent. Students completing feedback in paper form signed a paper-based consent form that was submitted separately to the questionnaires and stored securely by a single researcher (KB) who was not involved in the data analysis.

### Data analysis

Data were gathered from the students’ responses to the questions on the feedback forms and subsequently entered into Microsoft Excel. Qualitative data from the open-ended questions were extracted and imported into Atlas.ti. 22.2.4 for thematic coding. Although most qualitative responses comprised only a few sentences, as many qualitative data analysis steps as possible were adhered to [17]. Two researchers (QL and MYC) first immersed themselves in the data through repeated readings performed independently. An abductive analytical approach was employed, combining both deductive and inductive methods. The deductive analysis used categories from the feedback form as initial themes, whereas the inductive approach allowed for the emergence of new insights and unexpected patterns from the data. The relevant sections of the text were highlighted and assigned labels. These initial codes were analysed for similarities and grouped into themes. This was followed by a process of reviewing, refining themes, and naming themes. The two researchers engaged in regular discussions following independent analysis throughout the iterative process, which helped in identifying and mitigating potential biases and broadening interpretative perspectives. Any disagreements were resolved by discussion with a third reviewer (KB) until a consensus was reached. Finally, a report was produced to present a comprehensive argument that answered the research question. Exemplar quotes that reflected participants’ collective responses were extracted to illustrate each main theme.

## Results

### Sample description (PT and Speech)

The study included three cohorts: 47 third-year BSc Physiotherapy students in 2021, 46 fourth-year BSc Physiotherapy students in 2022, and 26 third-year B of Speech-Language and Hearing Therapy students in 2023. The response rates varied: 100% and 43% of the 2021 and 2022 cohorts, respectively, responded to at least one questionnaire administered throughout the module, and 38% of the 2023 cohort completed at least one survey on the selected topics to which they were exposed (Table 3).

**Table 3:** Response rates and demographic characteristics of the students in the module feedback surveys

Total enrolled students	% women	Mean (SD) age, years	Number of respondents (% of total)
BSc Physiotherapy III (2021), n = 47	85%	22.5 (2.4)	47 (100%)
BSc Physiotherapy IV (2022), n = 46	85%	23.0 (2.4)	20 (43%)
B of Speech-Language and Hearing Therapy III (2023), n = 26	100%	21.8 (0.8)	10 (38%)

### Main findings

Three main themes arose from the analysis of the participants’ responses. Descriptions of the respondents’ characteristics are provided for each exemplary quote, i.e., the participant number (e.g., P23) and the cohort (e.g., PT, year 4 for BSc Physiotherapy IV [2022]).

### Engagement and communication

#### Interaction with course coordinators

Most of the participants valued the level of support they received from the course coordinators. The students particularly commented on the prompt responses by the course coordinator, which reflected care and support, which seemed to have been a core need given that the mode of delivery was online.

*“I enjoyed the fact that any queries we had were always answered promptly and kindly. This definitely helped facilitate our engagement with the module.”* (P14, PT, year 4).

Many students reflected on the meaningfulness of the support they received. The nature and meaning of the support reflect the overall quality of the online interaction between participants and the course coordinators. The comments from the students also suggest that their queries and questions were understood by the course coordinators.

*“The communication and the response time with the module coordinator was very prompt, and the feedback was always clear. Knowing that the lecturers were only an email away was reassuring and I loved how they constantly reminded us of upcoming assessments and sent out clear communication and were willing to hear our suggestions.”* (P27, PT, year 4).

One negative aspect is related to feedback on online activities. The students expected feedback on all activities and expressed disappointment when feedback was not provided. Communication with students regarding feedback on online activities appeared to have been inadequate.

*“We didn’t collaborate at all nor did we give feedback. We will receive feedback, but we may have benefited from an example answer after each question.”* (P5, SLHT, year 3).

*“There was no feedback provided up to this time. But the way that feedback will be projected should be efficient.”* (P4, SLHT, year 3).

*“Most of the activities have not provided or generated any feedback yet.”* (P7, SLHT, year 3).

#### Interaction with peers

After class, online discussions provided a platform for interaction, fostering connections within groups and easing communication. The module included compulsory online debriefing and check-in sessions following specific topics to help consolidate the information. In addition, groups organised their own sessions, primarily centred around project completion. Collaborative activities, such as those in the thesis group, facilitated idea sharing. However, outside of these instances, opportunities for further connection with peers were limited.

*“The fact that we could choose the groups we worked with. It is a long time to work with others, and I got along well with my group.”* (P18, PT, year 4).

*“We did a collaborative activity in our thesis group. However, there were no other opportunities to connect and share ideas.”* (P5, SLHT, year 3).

#### Interaction with research project supervisors

The online research methodology course included a new approach for students to conduct their undergraduate research projects. This was a new experience for all the research project supervisors. The students expressed frustration with the misalignment between the guidance received from supervisors and what the module expected. The need for improved communication on the structure and processes of the renewed research methods course between the course coordinators and the research project supervisors to assist students optimally was emphasised.

*“The supervisors also need to be on par with what’s happening throughout the module; our supervisor asked us what needs to be done and weren’t acquainted with the template, which the rubric was marked according to, so we were conflicted with what our supervisor wanted and what the rubric/module wanted.”* (P4, PT, year 4).

### Content

#### Clear outcomes

The students provided contrasting reviews on the clarity of learning objectives and outcomes. Since the course was presented online, the clarification of learning outcomes instilled confidence in some students regarding expectations.

*“I enjoyed that each week was taken in phases and stages, and the outcomes for that week were made clear.”* (P17, PT, year 4).

Other students expressed a disconnect between the content and the outcomes of the research module, leading to a perception of redundancy or irrelevance.

*“Being honest, I did not really enjoy research. I think the main reason is that I did not know what the end product would be and would look like. We had all the classes, but it was not very meaningful to me as someone who has never done research for a thesis before and not knowing what we are working towards other than producing a case study and literature review.”* (P20, PT, year 4).

#### Workload

Some students perceived the workload to be excessive. This may have been due to the complexity of some tasks compounded by a lack of proper scaffolding, planning and communication from the faculty.

*“The amount of work that we went through was quite a lot and found it frustrating, which overwhelmed me a bit.”* (P4, SLHT, year 3).

*“There were also a few instances where the communication from the university’s side was lacking. This led to miscommunication; thus, the workload of the students increased to fix the problems created with this bad communication.”* (P23, PT, year 4).

Most students were able to complete the tasks in time and felt that the information was sufficient.

*“I had more than enough time.”* (P3, SLHT, year 3).

*“I was able to complete the tasks in time.”* (P7, SLHT, year 3).

#### Relevance

Most students felt that the content was directly relevant and useful in providing a comprehensive understanding of research methodologies and concepts.

*“The content was very helpful, especially in completing assignments; it was [well] explained, and no further research on how to do research needed to be done.”* (P16, PT, year 4).

Some feedback indicated a need for the research module to be regularly reviewed to ensure that all aspects were current. In the context of a rapidly evolving field such as research, especially during and after significant global events, it is crucial for content to be updated.

*“Some of the activities were outdated (e.g., the COVID masks).”* (P5, SLHT, year 3).

#### Logical step-by-step approach

Teaching research methodology is often challenging since students often find abstract concepts difficult to understand. Thus, the quantity and manner in which research content is delivered is an important consideration. The students enjoyed the step-by-step manner in which the content was presented, as it arguably allowed understanding and consolidation of one construct at a time and illustrated how the different research aspects are interlinked.

*“The order was logical and helped form my ideas from the foundation up, organised structure and there was sufficient time in my timetable to complete all the work.”* (P5, SLHT, year 3).

They valued the time to refresh and revisit materials, noting that the logical order helped build knowledge progressively.

*“I enjoyed the layout of the module, as it was clear and nicely laid out. We always knew what we had to do and how.”* (P11, PT, year 4).

*“I liked how it was set up on SUNLearn. It was easy to follow, and all the resources were easily found and accessible.”* (P5, PT, year 4).

#### Understanding the bigger picture

The students enjoyed the different activities and step-by-step nature in which the material was presented. However, they struggled to understand how all the different tasks were interrelated and how all the activities and skills would culminate in a final research project report. They especially commented on the need to have a comprehensive overview of the module, which may help them understand the importance of each of the online tasks.

*“It would be helpful to have an idea of the end product of the thesis at the beginning of the module to ensure the students knew what to work towards. This year, little information was given at various times, so we did not know what we were working towards.”* (P23, PT, year 4).

*“The module presented how to do our final research presentation in small pieces. However, it felt like there was no big picture. We were doing all these little tasks but could not understand how they fit together. When the final research project was completed, I could look back and finally see how all the little things came into place and played a part in the big project. I think it would be nice to know from the beginning what the final project consisted of and how we would get there. Big picture and then moving into the little pieces.”* (P2, PT, year 4).

#### Practical activities

The practical online activities provided students with an opportunity to ascertain how well they understood the content in a manner conducive to learning. The students perceived the practical exercises to be engaging, which also helped them appreciate the importance of certain elements in research. The students reported that the activities were relevant and engaging.

*“I found Activity 1 very interesting and a good way to practice what has been taught.”* (P6, SLHT, year 3).

*“Because you had to apply your knowledge in every activity. You had to have an understanding of the work, which helped.”* (P4, SLHT, year 3).

The students believed that critical thinking was encouraged, fostering a deeper level of engagement and learning.

*“I learned more about what critical thinking actually means, and the accompanying activity helped us test our knowledge thereof.”* (P4, SLHT, year 3).

Engaging in practical work aided in comprehension, particularly after reviewing the recordings.

*“Many concepts I was familiar with already, but I also learned and understood many new terms and concepts, which was helpful.”* (P4, SLHT, year 3).

#### Linkage to professional practice

Many of the learners highlighted the necessity of understanding the importance of research to rehabilitation practice. In addition, the course activities and research project were intertwined with clinical practice. Some of the students commented that the module helped them adopt a more patient-centred approach through the development of skills such as critical thinking and problem solving.

*“It afforded me the opportunity to have a more patient-centred approach when analysing literature and planning for future studies.”* (P19, PT, year 4).

Traditionally, research methodology courses focus exclusively on research methodology content. Considering the difficulty of students grasping the relevance of research parallel to their undergraduate professional training, this new online research methodology course also included information on the relevance of research to rehabilitation practice.

*“Learning about the importance of research in rehabilitation according to other students in the discussion forum, as well as Activity 1.”* (P6, SLHT, year 3).

### Online user-interface

#### Navigation

Virtually all the students commented on the importance of user-friendliness. They commented that the online organisation of the content was well designed. Positive feedback was given for the simplicity of the layout and the relevance of information on the SUNLearn page.

*“I loved the fact that the SUNLearn page was so organised.”* (P7, PT, year 4).

*“I enjoyed the layout of the module, as it was clear and nicely laid out. We always knew what we had to do and how.”* (P11, PT, year 4).

#### Design elements

The participants reported that the lecture podcasts were informative, whereas some desired more specific content. The participants found the lecture podcasts to be of good quality. However, they suggested podcasts that are more visually stimulating to facilitate learning.

*“It was good quality, with detailed information.”* (P4, SLHT, year 3).

*“Some of the videos at the beginning of the module were a bit repetitive, and I felt like they did not always teach me anything new, but I know that the foundation had to be laid regarding certain concepts.”* (P9, PT, year 4).

They also noted the volume and monotonous tone of the lecturer voices on the podcasts as important considerations for improving student engagement.

*“The lecturer spoke slow and monotone. It was very difficult to stay focused.”* (P8, SLHT, year 3).

#### Duration and timely availability of learning materials

The students had mixed reviews on the duration of the podcasts. Some perceived the podcast as lengthy, resulting in feeling bored and limited learning, whereas others viewed the duration as adequate. However, an optimal time for podcasts was not suggested.

*“They were filled with valuable information and were condensed into an appropriate amount of time.”* (P5, SLHT, year 3).

*“Make the podcasts shorter and more visually stimulating. Break the sections into individual podcasts.”* (P8, SLHT, year 3).

Students experienced the timely availability of research, which should be well coordinated with the scheduling of specific topic sessions. Students suggest whether it would be beneficial to have access to materials well ahead of scheduled sessions to review content prior to the online session. Additionally, continued access to previous materials enabled the students to clarify or review information relevant to their current coursework.

*“Also keeping the third-year module available on SunLearn would be appropriate. There were times I wanted to access something on SunLearn, but because we could not access SunLearn anymore from previous years, I could not access information from the third-year section of the module.”* (P11, PT, year 4).

#### Technical issues

The students mentioned that it was frustrating when links were inactive because it prohibited them from accessing the learning material. They also suggested easier access to the online presentations, as they often had to search for relevant presentations. Interestingly, none of the students had issues with internet connectivity.

*“Not being able to click on the YouTube links of the videos.”* (P2, SLHT, year 3).

As seen in the results, there are mixed views on the content and user friendliness of the undergraduate online research methodology course. The students also indicated a need for improved communication and increased peer and supervisor contact throughout the course.

## Discussion

Teaching research methodology to undergraduate students is often complex, particularly in disciplines like rehabilitation, where students primarily engage in clinical training. The shift towards online education provides new and innovative avenues to enhance the teaching and learning of research methodology. To our knowledge, this is the first study to explore the perspectives and experiences of rehabilitation students with a fully online research module which was delivered for the first time during the COVID-19 pandemic. Rehabilitation undergraduate students commonly experience research methodology as a complicated and challenging topic. Therefore, considering the increased use of online education, it is important for educators in health science to gain insights into the planning and delivery of online research methodology courses. Our findings provide new insights for online research methodology course planning specifically as it pertains to rehabilitation students (Table 4).

**Table 4:** Unique contributions/recommendations for online research methodology for rehabilitation students

Purpose-driven quality course coordination that is intentionally designed for an online research methodology course			
Multi-stakeholder support mechanisms that integrate collaboration between educators, course coordinators and peers			
Co-designed information and communication strategies developed in collaboration with all stakeholders			
Course structural planning that includes explicit visualization of the final research product			
User-friendly online platform tailored to the specific needs of rehabilitation students to facilitate learning and engagement			

A key finding from our research is that while the rehabilitation students valued dedicated, timely and meaningful support from course coordinators, such support needs to be intentionally structured to optimise the online learning experience and ensure that graduate competencies are met. Rehabilitation education typically relies on face-to-face learning, given the hands-on nature of clinical practice. This adds complexity to the transition to the online teaching of research methodology. To address these challenges, we suggest that online research courses in rehabilitation must be thoughtfully designed to incorporate robust support mechanisms that bridge the physical gap between instructors and students. This support is crucial for the success of an online research course since without physical proximity, students may feel isolated or disconnected. Designers of online research courses should therefore incorporate adequate and effective strategies that foster optimal student support. This aligns with previous research emphasising the heightened need for instructor presence in online education [18].

The course coordinators should ensure that the online course environment is safe, learning friendly, and supportive. The students in our study appreciated the prompt and useful responses from coordinators, which not only resolved their queries but also made the students feel that the course coordinators cared about their engagement in the course. This support was particularly important, as fostering student engagement proved to be more challenging in the online course setting than in face-to-face courses. Course coordinator support should be augmented by peer-driven support mechanisms that involve well-coordinated multi-dimensional channels of support. Strategies such as purpose-driven activities, collaborative projects and discussion forums enabled the students to build connections and support others’ learning. This approach would be innovative in replicating the support network typically available in face-to-face settings to better meet the specific needs of teaching rehabilitation students. Educators planning to offer online research methodology courses should thus advocate for sufficient resources to meet the associated student support demands [19]. Effective engagement by course coordinators positively impacts the overall quality of online interaction and communication, especially since a high premium is placed on asynchronous learning and teaching methods and self-directed learning.

Students often leverage their learning on effective feedback. The students in our study felt that there was a shortcoming regarding expectations about feedback on online activities. Students expected feedback on all activities as reassurance of their work or, at the very least, clear communication when feedback would not be provided. Regular feedback may be linked to the self-driven nature of the online module, as students seek connectedness with the educator and material [1]. Communication about feedback helps students put measures in place to check their understanding of tasks or outcomes [20]. These findings emphasised the importance of communication and processes within the module. Appropriate feedback is essential in an online setting to help students gauge their progress and understand areas needing improvement [21]. A dedicated person may be needed to provide timely feed-back to the rehabilitation students. Alternative feedback pathways, such as automated feedback for certain tasks, peer feedback sessions or guided self-assessment tools, could be used to complement educator feedback [21]. Our findings also suggest that feedback mechanisms for online research programs should be tailored to meet student-centered needs.

The need for clear communication between students and course coordinators is amplified in online research methods courses for rehabilitation. Considering that students find research methodology a complex subject, well-designed communication methods can improve student learning and experience. However, the heightened need for communication may put a strain on educators’ time and resources. While we do not have a well-defined communication and information strategy for face-to-face research methods teaching, it is essential that these strategies be co-designed with all stakeholders (including students and educators). These strategies should be clearly communicated and agreed upon before the course begins. Human resource implications for effective communication with students should therefore be considered when an online research methods course is rolled.

We also found that communication between educators is key for well-perceived research methods in online courses. Undergraduate research in rehabilitation is typically an all-department effort (involving PT, OT and SLTH), with each or most faculty contributing to varying capacities. In our context, the key academic players were the course coordinators and research supervisors, who act as research mentors to students. Unfortunately, the students reported frustration, possibly due to a lack of clear guidance and adaptation to the new methodology/course. The communication challenges between course coordinators and supervisors created confusion about the next steps of their research journey. Risk mitigation strategies could include a clear communication plan or a shared online log where research supervisors can document issues or complaints. This finding also highlights that research supervisors require significant levels of support when a new research approach is introduced. Developers of online research methodology courses should develop a support strategy for supervisors and supervisor‒student dyads to ensure that all parties that contribute to student learning feel supported and are on the same page while maintaining students’ autonomy [22]. Additionally, the utilisation of postgraduate students and postdocs as mentors may provide further channels for guidance in the technical aspects of research projects for which faculty supervisors may not have time [23].

Providing clear learning objectives and outcomes can support good communication between educators. The students in our study commented on the importance of clear learning objectives and outcomes in focusing their efforts and understanding the relevance of the comprehensive material. The positive feedback from students emphasises the importance of clear communication in online research methodology education. Clear objectives help build student confidence and provide a roadmap for their learning journey. Clear outcomes that emphasize how research methods are applied in clinical settings can help rehabilitation students see the practical value of the content, making it more engaging and relevant to their future careers. According to the literature, providing explicit learning goals reassures students by clarifying what is expected of them and providing guidance on how to achieve the goals [24]. Online research methodology course developers should therefore make an intentional effort and pilot draft outcomes related to online engagement to ensure that students are well informed about this foundational aspect of course design.

In a face-to-face class environment, students often interact unintentionally. Peer interaction in an online environment can be challenging unless specific measures are put in place to support peer interactions. The research shows that after-class online discussions and collaborative activities, such as those within thesis groups, were effective in fostering connections and facilitating idea sharing. These structured peer interactions helped create a sense of community among students and eased communication barriers. However, outside these formal activities, opportunities for further peer interaction were limited. This limitation in peer engagement is a common challenge in online learning environments [25]. The students indicated that this aspect should be improved. Future online research methodology course developers should consider incorporating additional unstructured peer interaction opportunities such as online discussion forums, group chats or virtual meetups, allowing students to connect more organically to enhance their learning experience. Unstructured peer interaction opportunities by creating informal space for students to meet within thesis groups or classes to discuss their research project might facilitate increased peer interactions [26].

The teaching of research methodology in rehabilitation contexts can be challenging due to the abstract nature of the content. Undergraduate rehabilitation students often felt that they do not understand the research process or why it is incorporated into their online course. To address this, we opted for a step-by-step approach, gradually developing and cementing new skills. This structured approach aligns with best practices in instructional design for online learning [27], particularly for complex subjects such as research methodology. The students provided positive feedback on this approach, possibly because it allowed them to understand and consolidate complex concepts incrementally, reinforcing the interconnectedness of different research components.

Despite appreciating the structured approach, the students struggled to see how individual tasks connected to the final research project. This indicates a need for more explicit visual representations of the end product and how different tasks integrate. Such representations can help students understand the overall process and the importance of each task. Integrating research methodology with professional practice, particularly in rehabilitation, was highly valuable. This approach bridges the gap between theoretical knowledge and real-world application [28, 29], making the course content more meaningful. For example, enhanced digital literacy and communication skills prepare students for continued multidisciplinary collaboration through digital platforms [30], which is particularly important for those working in remote underserved areas. Additionally, online courses often require students to take greater responsibility for their learning, fostering self-directed learning skills, which are crucial for lifelong learning, as practitioners need to keep abreast of current evidence-based care [30]. Students thus appreciated the relevance of research skills acquired in a virtual context to their future contemporary professional roles, which enhanced their motivation and engagement.

The user interface and design elements of the online platform played crucial roles in shaping the students' learning experiences. The simplicity and user friendliness of the interface were positively perceived by the students. A well-organised interface helps students easily navigate through course materials, reducing frustration and enhancing the learning experience. Positive feedback on the design and layout indicates that a clear and intuitive user interface is vital for successful online education, in alignment with previous research emphasising the importance of intuitive and well-organised online learning environments [25]. However, the effectiveness of lecture podcasts received mixed reviews. While lecture podcasts were informative, the students occasionally found them monotonous and too lengthy. Students found very lengthy podcasts unengaging but did not specify an optimal duration. This suggests a need for more engaging, concise and visually stimulating content, as well as consideration of the optimal podcast duration. One review reported the common length of podcasts in medical education to be between 15 and 20 min, which was found to be consistent with reported listener preference [31]. Dynamic presentation styles and varying content delivery methods can keep students engaged and facilitate better learning [32]. Chunking content into smaller, more manageable segments can help maintain student attention and motivation [27] but may also perpetuate fragmentation. To combat this, the use of concept maps, or the provision of such maps at the beginning or end of the course, is effective in illustrating the relatedness of concepts.

The timely availability of learning materials was another key factor appreciated by the students, as it allowed for adequate preparation and review. This highlights the importance of providing resources well ahead of scheduled sessions, ensuring that students have ample time to engage with the content at their own pace and convenience. However, technical challenges, such as inactive links and difficulty accessing presentations, frustrated the students. These issues underscore the need for robust technical support and regular maintenance of online platforms to ensure seamless access to learning resources. Addressing such technical problems promptly and proactively can significantly enhance the overall learning experience and minimise disruptions to students' learning journeys.

### Limitations

While this study provides valuable insights into rehabilitation students' experiences, it is limited by its focus on a limited year groups from a single university’s rehabilitation department in South Africa. Future research could explore these themes across different contexts, e.g., institutions or disciplines, to increase the generalizability of the findings. Feedback from multiple cohorts will be needed for affirmative guidance on the development of online research methodology courses.

A potential limitation of this study is the classification of third- and fourth-year physiotherapy students as separate cohorts, despite being the same group of students progressing through the research methodology curriculum. Although new content was introduced in the fourth year, the possibility of retrospective recall may have influenced students' perceptions of the module. This overlap could impact how students experienced and evaluated the course components across different years, potentially affecting the interpretation of their feedback. Future studies could explore longitudinal designs to assess how perceptions evolve over time within the same student group.

Future research should include more in-depth qualitative analyses to provide further insight into student perspectives to inform the design of online research methods.

An assessment of collaborative interprofessional learning of the module as an avenue to foster multidisciplinary practice should also be explored. The long-term impact of online courses on practicing graduates will also be useful.

Further quantitative research could quantify the effect of the online research methodology course. A rigorous assessment of the far-ranging impacts of the course on students’ research experiences and outcomes, including institutional funding, knowledge creation, and student retention in postgraduate research, is warranted [33].

## Conclusion

The findings underscore several critical aspects of effective online education. Strong, supportive communication from course coordinators, a clear and structured presentation of content aligned with explicit outcomes for each learning session, and practical, engaging activities are pivotal in enhancing the online learning experience. Additionally, the integration of professional practice relevance and intuitive online interfaces significantly contributes to student satisfaction and learning outcomes. Addressing the identified gaps, such as the need for more systematic feedback and improved clarity on the integration of tasks, can further refine and optimise online educational offerings and may contribute to the development of scholarship as key graduate attribute of undergraduate rehabilitation sciences students at South African universities.

## Supplementary Information

Supplementary Material 1

## Data Availability

The datasets generated and/or analysed during the current study are not publicly available due to privacy restrictions of SUNLearn and coursework copyright vesting in Stellenbosch University but are available from the corresponding author on request.

## Abbreviations

CTL: Centre for Teaching and Learning
PT: Physiotherapy
RM 372/472: Research Methods 372/472
SPH: Speech Language and Hearing Therapy
SU: Stellenbosch University
