# Supplementary material for: Redesigning online research methodology education: insights from undergraduate rehabilitation students' experiences in South Africa
Source: BMC Med Educ. 2025 Mar 31;25:463. doi: 10.1186/s12909-025-06934-0 (PMC11956173; doi:10.1186/s12909-025-06934-0)
Supplement: Supplementary file 1 — Supplementary Material 1 [file 12909_2025_6934_MOESM1_ESM.pdf]

[redacted] [redacted] [redacted] [redacted] [redacted] [redacted]

2021 RM 372 Student Feedback Form (15 & 16 July)

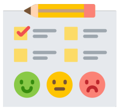

Page 1

Overall impressions

1 * What did you enjoy most about the past 2 days' Research Methods and why?

Paragraph

Path: p

2 * What did you find most frustrating and why?

Paragraph

Path: p

3 Any additional comments on the past 2 days - including ideas on how we may improve?

Paragraph

Path: p

Session: Rehabilitation Professionals: Researchers by profession

4 * Please **rate** the session "**Rehab professionals - Researchers by profession**" by giving it a mark from 0-10 ("0" meaning you gained nothing from the session versus "10" meaning you learnt maximally from the session). Please **motivate** your answer.

5 * I found the topic/session interesting.

	strongly disagree	somewhat disagree	neutral	somewhat agree	strongly agree
Please rate your agreement:	○	○	○	○	○

6 * The information/concepts in the session was easily understandable.

	strongly disagree	somewhat disagree	neutral	somewhat agree	strongly agree
Please rate your agreement:	○	○	○	○	○

7 Any additional comments on this session?

Page 2

Session: Health Science Research - Understanding the past to shape the future

8 * Please **rate** the topic/session "**Health Science Research - Understanding the past to shape the future**" by giving it a mark from 0-10 ("0" meaning you gained nothing from the session versus "10" meaning you learnt maximally from the session). Please **motivate** your answer.

9 * I found the topic/session interesting.

	strongly disagree	somewhat disagree	neutral	somewhat agree	strongly agree
Please rate your agreement:	○	○	○	○	○

10 * The information/concepts in the session was easily understandable.

	strongly disagree	somewhat disagree	neutral	somewhat agree	strongly agree
Please rate your agreement:	○	○	○	○	○

11 Any additional comments on this session?

Page 3

Session: Systematic review - Vehicle for EBM

*

12 Please **rate** the topic/session "**Systematic Review - Vehicle for EBM**" by giving it a mark from 0-10 ("0" meaning you gained nothing from the session versus "10" meaning you learnt maximally from the session). Please **motivate** your answer.

13 * I found the topic/session interesting.

	strongly disagree	somewhat disagree	neutral	somewhat agree	strongly agree
Please rate your agreement:	○	○	○	○	○

14 * The information/concepts in the session was easily understandable.

	strongly disagree	somewhat disagree	neutral	somewhat agree	strongly agree
Please rate your agreement:	○	○	○	○	○

15 Any additional comments on this session?

Page 4

Session: EBM to EBP to EBR

16 * Please **rate** the session "**EBM to EBP to EBR**" by giving it a mark from 0-10 ("0" meaning you gained nothing from the session versus "10" meaning you learnt maximally from the session). Please **motivate** your answer.

17 * I found the topic/session interesting.

	strongly disagree	somewhat disagree	neutral	somewhat agree	strongly agree
Please rate your agreement:	○	○	○	○	○

18 * The information/concepts in the session was easily understandable.

	strongly disagree	somewhat disagree	neutral	somewhat agree	strongly agree
Please rate your agreement:	○	○	○	○	○

19 Any additional comments on this session?

Page 5

Session: Understanding equity in health research

20 *

Please **rate** the session "**Understanding equity in health research**" by giving it a mark from 0-10 ("0" meaning you gained nothing from the session versus "10" meaning you learnt maximally from the session). Please **motivate** your answer.

21 * I found the topic/session interesting.

	strongly disagree	somewhat disagree	neutral	somewhat agree	strongly agree
Please rate your agreement:	○	○	○	○	○

22 * The information/concepts in the session was easily understandable.

	strongly disagree	somewhat disagree	neutral	somewhat agree	strongly agree
Please rate your agreement:	○	○	○	○	○

23 Any additional comments on this session?

Close this window

[redacted]

2021 RM 372 Day 1 Student Feedback Form

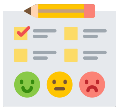

Page 1

Overall impressions

1 * How do you feel about Research after completing Day 1 of the module?

	completely uninterested	mostly uninterested	somewhat uninterested	neutral	somewhat interested	mostly interested	very interested
Rate your level of interest:	○	○	○	○	○	○	○

2 * The teaching and learning activities contributed to my understanding of Research Methods and the application thereof in Rehab Sciences.

	Strongly disagree	Somewhat disagree	Neutral	Somewhat agree	Strongly agree
Choose the best option:	○	○	○	○	○

3 * How confident are you in **applying** what you have learned today?

	not confident at all	somewhat unsure	somewhat confident	very confident
Rate your level of confidence:	○	○	○	○

4 What is one thing that you enjoyed the most today and why?

5 * What did you find to be the most frustrating and why?

6 * Was the **order** and/or **structure** and/or **time allocation** of the different sessions (including breaks) beneficial for you to be able to fully participate and/or complete tasks? Why do you say that?

7 * Did you require more information in the initial introductory session to the module on Teams? If so, what sort of information would you have liked to hear about?

8 * The learning objectives of each topic (Orientation, Critical thinking and Story Time) were clear to me.

	strongly disagree	somewhat disagree	somewhat agree	strongly agree
Rate your agreement with the statement:	○	○	○	○

9 * What did you think of the lecture podcasts (e.g. would have liked more/less detail/depth/time, good quality, etc.).

10 * What did you think of the number of **YouTube videos** that were provided?

- ○ too much
- ○ too little
- ○ just right

11 Any additional comments on the videos - e.g. type, length etc.? (optional)

12 * What did you think of the number of **practical activities** (e.g. scenarios, quizzes) that were provided?

- ○ too much
- ○ too little
- ○ just right

13 * Did the content and nature of the activities allow you to practice and better understand what you have learned about in the session? Briefly explain your answer.

14 * Did you learn from your classmates/group members today?

○ Yes ○ No

15 * Did you find the way that **feedback** was provided on various activities sufficient? If not, please explain and give suggestions for improvement.

Page 2

Topic 1: Orientation to Research in Rehabilitation

16 * Please **rate** the topic/session "**Orientation to Research in Rehab**" by giving it a mark from 0-10 ("0" meaning you gained nothing from the session versus "10" meaning you learnt maximally from the session). Please **motivate** your answer.

17 * I found the topic/session interesting.

	strongly disagree	somewhat disagree	neutral	somewhat agree	strongly agree
Please rate your agreement:	○	○	○	○	○

18 * The information/concepts in the session was easily understandable.

	strongly disagree	somewhat disagree	neutral	somewhat agree	strongly agree
Please rate your agreement:	○	○	○	○	○

19 Any additional comments on this session?

Page 3

Topic 2: Critical thinking and problem-solving in research

20 * Please **rate** the topic/session "**Critical thinking and problem-solving in research**" by giving it a mark from 0-10 ("0" meaning you gained nothing from the session versus "10" meaning you learnt maximally from the session). Please **motivate** your answer.

21 * I found the topic/session interesting.

	strongly disagree	somewhat disagree	neutral	somewhat agree	strongly agree
Please rate your agreement:	○	○	○	○	○

22 * The information/concepts in the session was easily understandable.

	strongly disagree	somewhat disagree	neutral	somewhat agree	strongly agree
Please rate your agreement:	○	○	○	○	○

23 Any additional comments on this session?

Page 4

Afternoon session: Story time (Project-related topic 1)

24 * Please **rate** the topic/session "**Story time**" by giving it a mark from 0-10 ("0" meaning you gained nothing from the session versus "10" meaning you learnt maximally from the session). Please **motivate** your answer.

25 * I found the topic/session interesting.

	strongly disagree	somewhat disagree	neutral	somewhat agree	strongly agree
Please rate your agreement:	○	○	○	○	○

26 * The information/concepts in the session was easily understandable.

	strongly disagree	somewhat disagree	neutral	somewhat agree	strongly agree
Please rate your agreement:	○	○	○	○	○

27 Any additional comments on this session?

Page 5

Engaging with the course material / online experience

28 * Did you find the course page scavenger hunt useful?

	not useful	somewhat	very useful
Please rate the usefulness of the activity:	○	○	○

29 * What did you think of the layout and design of the SUNLearn page (e.g. too busy, easy to navigate, confusing, too many links, etc)?

30 * How did you access the course (laptop / mobile device)? Did you have any concerns regarding data usage (e.g. when leaving SUNLearn to link to YouTube)?

31 * Did you find it easy to connect with other students (group work, sharing resources, ideas)? Why or why not?

Page 6

Final thoughts

32 Do you have any other comments/feedback for this module? (optional)

Close this window

[redacted]

2021 RM 372 Final Course Evaluation Student Feedback Form

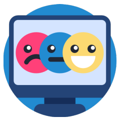

Page 1

For each of the following questions, please tick the option that best describes your experience
Vir elk van die volgende vrae, merk asseblief die opsie wat jou ervaring die beste beskryf

1 * How do you feel about **Research** after completing the RM 372 module?
*Hoe voel jy oor **Navorsing** noudat jy die NVM 372 module voltooi het?*

	completely uninterested/ stel glad nie belang nie	mostly uninterested/ stel meestal nie belang nie	somewhat uninterested/ effens onbelangstellend	neutral/ neutraal	somewhat interested/ stel effens belang	mostly interested/ stel meestal belang	very interested/ stel baie belang
Rate your level of interest:	○	○	○	○	○	○	○

2 * The **pace** of the RM 372 module was ...
*Die **tempo** in die NVM 372 module was ...*

	too slow/te stadig	just right/net reg	too fast/te vinnig
Rate your response:	○	○	○

3 * The number of **synchronous** sessions was ...
*Die hoeveelheid **sinkroniese** sessies was ...*

	too few/te min	just right/net reg	too many/te veel
Rate your response:	○	○	○

4 * The **learning outcomes** for this module were **available and easily accessible**.
*Die **leeruitkomste** vir hierdie module was **beskikbaar en maklik toeganklik.***

	strongly disagree/ stem glad nie saam nie	disagree/ verskil	neutral/ neutraal	agree/ stem saam	strongly agree/ stem beslis saam
Rate your agreement with the statement:	○	○	○	○	○

5 * The **learning outcomes directed** my learning, and it was **clear** what I had to learn/achieve at the end of a session.
*Die **leeruitkomste** het **gerigtheid** aan my leer gegee, en dit was **duidelik** wat ek moes leer/bereik teen die einde van 'n sessie.*

	strongly disagree/ stem glad nie saam nie	disagree/ verskil	neutral/ neutraal	agree/ stem saam	strongly agree/ stem beslis saam
Rate your agreement with the statement:	○	○	○	○	○

6 * The module **content** was presented in a manner that made it **easy** for me to understand the content and that **facilitated** my learning.

*Die module-**inhoud** is op so 'n manier aangebied dat dit vir my maklik was om die inhoud te **verstaan** en dat dit my leer **gefasiliteer** het.*

	strongly disagree/ stem glad nie saam nie	disagree/ verskil	neutral/ neutraal	agree/ stem saam	strongly agree/ stem beslis saam
Rate your agreement with the statement:	○	○	○	○	○

7 *

All prescribed **study material** (articles/ reading material, online resources, podcasts and lecture notes, YouTube videos, etc.) **facilitated my understanding of the content**.
*Alle voorgeskrewe **studiemateriaal** (artikels/ leesmateriaal, aanlyn-bronne, potgooie en lesingnotas, YouTube videos ens.) het my **begrip van die inhoud gefasiliteer.***

	strongly disagree/ stem glad nie saam nie	disagree/ verskil	neutral/ neutraal	agree/ stem saam	strongly agree/ stem beslis saam
Rate your agreement with the statement:	○	○	○	○	○

8 *

The practical learning **activities** (individual and group), e.g. forum posts, scenarios, writing tasks, etc., enabled me to achieve the learning outcomes.
*Die praktiese **leeraktiwiteite** (individueel en groepsverband), bv. forumplasings, scenarios, skryftake, ens., het my in staat gestel om die leeruitkomste te bereik.*

	strongly disagree/ stem glad nie saam nie	disagree/ verskil	neutral/ neutraal	agree/ stem saam	strongly agree/ stem beslis saam
Rate your agreement with the statement:	○	○	○	○	○

9 *

I found the teaching and learning material (podcasts, activities, etc.) **interesting and fun**.
*Ek het die leer en onderrig materiaal (potgooie, aktiwiteite, ens.) **interessant en prettig** gevind.*

	strongly disagree/ stem glad nie saam nie	disagree/ verskil	neutral/ neutraal	agree/ stem saam	strongly agree/ stem beslis saam
Rate your agreement with the statement:	○	○	○	○	○

10 *

I **engaged** with learning activities **online** (SUNLearn packages, Teams meetings) ...
*My **deelneming** aan **aanlyn** leeraktiwiteite (SUNLearn-pakkette, Teams-vergaderings) kan soos volg beskryf word ...*

	never/ nooit	rarely/ skaars	sometimes/ soms	often/ gereeld	always/ altyd
Rate your agreement with the statement:	○	○	○	○	○

11 *

I prefer keeping the RM module as a **fully online** offering in the future.
*Ek verkies dat die NVM module as a **volledige aanlyn-aanbieding** gehou word in die toekoms.*

	strongly disagree/ stem glad nie saam nie	disagree/ verskil	neutral/ neutraal	agree/ stem saam	strongly agree/ stem beslis saam
Rate your agreement with the statement:	○	○	○	○	○

12 *

A **big responsibility rests with me** to ensure that I achieve the outcomes of each of the RM 372 module sessions.
*Daar berus 'n **groot verantwoordelikheid by my** om te sorg dat ek die uitkomste van elk van die NVM 372 sessies bereik.*

	strongly disagree/ stem glad nie saam nie	disagree/ verskil	neutral/ neutraal	agree/ stem saam	strongly agree/ stem beslis saam
Rate your agreement with the statement:	○	○	○	○	○

13 *

Feedback on activities and assignments enabled me to **judge how well I was progressing**.
***Terugvoer** oor aktiwiteite en take het my in staat gestel om te **oordeel hoe goed ek vorder**.*

	strongly disagree/ stem glad nie saam nie	disagree/ verskil	neutral/ neutraal	agree/ stem saam	strongly agree/ stem beslis saam
Rate your agreement with the statement:	○	○	○	○	○

14 *

Feedback on activities and assignments was given **promptly**.
***Terugvoer** oor aktiwiteite en take is **stiptelik** deurgegee.*

	strongly disagree/ stem glad nie saam nie	disagree/ verskil	neutral/ neutraal	agree/ stem saam	strongly agree/ stem beslis saam
Rate your agreement with the statement:	○	○	○	○	○

15 * Before each **assessment**, it was made **clear** to me what would be **expected** from me in that assessment.
*Daar is voor elke **assessering** aan my **duidelik** gemaak wat in daardie assessering van my **verwag** sou word.*

	strongly disagree/ stem glad nie saam nie	disagree/ verskil	neutral/ neutraal	agree/ stem saam	strongly agree/ stem beslis saam
Rate your agreement with the statement:	○	○	○	○	○

16 * **Assessments** were always related to the **learning outcomes** for the RM 372 module.
***Assesserings** het altyd met die **leeruitkomste** vir die NVM 372 module verband gehou.*

	strongly disagree/ stem glad nie saam nie	disagree/ verskil	neutral/ neutraal	agree/ stem saam	strongly agree/ stem beslis saam
Rate your agreement with the statement:	○	○	○	○	○

17 * The **workload** in the RM 372 module was reasonable.
*Die **werkslading** in die NVM 372 module was redelik.*

	strongly disagree/ stem glad nie saam nie	disagree/ verskil	neutral/ neutraal	agree/ stem saam	strongly agree/ stem beslis saam
Rate your agreement with the statement:	○	○	○	○	○

18 * I found that **achieving the outcomes** of the RM 372 module was ...
*Ek het ondervind dat, om die **uitkomste van die NVM 372 module te bereik**, was ...*

	very difficult/ baie moeilik	difficult/ moeilik	neutral/ neutraal	easy/ maklik	very easy/ baie maklik
Rate the best option:	○	○	○	○	○

19 * How confident are you in **applying** what you have learned this past semester?

	not confident at all	somewhat unsure	somewhat confident	very confident
Rate your level of confidence:	○	○	○	○

20 * Did you learn from your **classmates/group members** during the module?

○ Yes ○ No

21 * The **language of tuition** in the RM 372 module was appropriate and as communicated to the class at the start of the module.
*Die **taal van onderrig** in die NVM 372 module was gepas en soos aan die klas gekommunikeer aan die begin van die module.*

	strongly disagree/ stem glad nie saam nie	disagree/ verskil	neutral/ neutraal	agree/ stem saam	strongly agree/ stem beslis saam
Rate your agreement with the statement:	○	○	○	○	○

Page 2

Open-ended questions
Oop-antwoord-vrae

22 What did you **like most** in the RM 372 module, and **why**?
*Waarvan het jy die **meeste gehou** in die NVM 372 module, en **hoekom**?*

23 What aspects of this module need to be **improved,** and **why**?
*Watter aspekte van hierdie module moet **verbeter** word, en **hoekom**?*

Page 3

Final thoughts

24 Do you have **any other comments/feedback** for the RM 372 module? (optional)
*Het jy **enige ander opmerkings/terugvoer** aangaande die NVM 372 module? (opsioneel)*

Close this window
